# An exceptional case of a huge pseudoaneurysm caused by an epicardial electrode

**DOI:** 10.11604/pamj.2023.46.22.40433

**Published:** 2023-09-14

**Authors:** Yassine Morjane, Alexandre Sebestyen

**Affiliations:** 1 Service de Chirurgie Cardiaque, Centre Hospitalier Universitaire Grenoble-Alpes, La Tronche, France

**Keywords:** Pseudoaneurysm, epicardial electrode, perforation

## Image in medicine

Our patient had a history of aortic valve replacement 18 years ago with a mechanical valve. After sternotomy, there is a huge pseudoaneurysm measuring 70 mm of the aortic root (A, B), totally adhering to the right atrium, which is pushed back and compressed. The aorta will be opened vertically downwards, allowing to see this enormous pseudoaneurysm encompassing the right coronary sinus (C). At this level, it exists a perforation of the aorta by an epicardial electrode and caused this pseudoaneurysm (D), which we had left in place 18 years ago to stimulate the right atrium. We finished the operation with a segment replacement by a Dacron tube.

**Figure 1:**
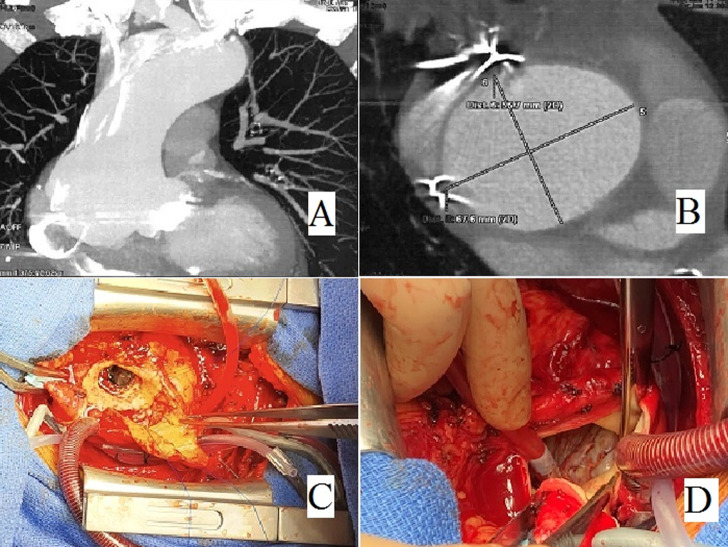
A) frontal section of a thoracic CT angiography showing a pseudoaneurysm of the tubular aorta; B) thoracic CT angiography in cross-section showing a 70 mm ectasia of the aortic root; C) intraoperative view showing a huge ectasia or pseudoaneurysm encompassing the right coronary sinus; D) intraoperative view showing perforation of the aorta by an epicardial electrode

